# Conservation of Monuments by a Three-Layered Compatible Treatment of TEOS-Nano-Calcium Oxalate Consolidant and TEOS-PDMS-TiO_2_ Hydrophobic/Photoactive Hybrid Nanomaterials

**DOI:** 10.3390/ma11050684

**Published:** 2018-04-27

**Authors:** Chrysi Kapridaki, Anastasia Verganelaki, Pipina Dimitriadou, Pagona Maravelaki-Kalaitzaki

**Affiliations:** School of Architecture, Technical University of Crete, Polytechnioupolis, Akrotiri, 73100 Chania, Crete, Greece; ckapridaki@gmail.com (C.K.); anastvergan@gmail.com (A.V.); pipina_dim@hotmail.com (P.D.)

**Keywords:** conservation, monuments, nanostructured, TEOS-nano-Calcium Oxalate, PDMS-SiO_2_-TiO_2_ nanocomposite, compatible, hydrophobic, self-cleaning, limestone, cement mortars

## Abstract

In the conservation of monuments, research on innovative nanocomposites with strengthening, hydrophobic and self-cleaning properties have attracted the interest of the scientific community and promising results have been obtained as a result. In this study, stemming from the need for the compatibility of treatments in terms of nanocomposite/substrate, a three-layered compatible treatment providing strengthening, hydrophobic, and self-cleaning properties is proposed. This conservation approach was implemented treating lithotypes and mortars of different porosity and petrographic characteristics with a three-layered treatment comprising: (a) a consolidant, tetraethoxysilane (TEOS)-nano-Calcium Oxalate; (b) a hydrophobic layer of TEOS-polydimethylsiloxane (PDMS); and (c) a self-cleaning layer of TiO_2_ nanoparticles from titanium tetra-isopropoxide with oxalic acid as hole-scavenger. After the three-layered treatment, the surface hydrophobicity was improved due to PDMS and nano-TiO_2_ in the interface substrate/atmosphere, as proven by the homogeneity and the Si–O–Ti hetero-linkages of the blend protective/self-cleaning layers observed by Scanning Electron Microscope (SEM), Transmission Electron Microscope (TEM) and Fourier-Transform Infrared Spectroscopy (FTIR). The aesthetic, microstructural, mechanical and permeabile compatibility of the majority of treated substrates ranged within acceptability limits. The improved photocatalytic activity, as proven by the total discoloration of methylene blue in the majority of cases, was attributed to the anchorage of TiO_2_, through the Si–O–Ti bonds to SiO_2_, in the interface with the atmosphere, thus enhancing photoactivation.

## 1. Introduction

Stone and mortars in monumental and contemporary architecture suffer from increasing weathering mainly attributed to anthropogenic activities, which induce dramatic climatic changes. The vulnerability of the construction materials to this enhanced environmental loading depends upon their intrinsic characteristics and the role entailed in the construction [1,2]. The protection of monumental cultural heritage with innovative nanocomposites is in the vanguard of conservation science and a plethora of research activities are dedicated to the design and validation of compatible nanomaterials that exhibit strengthening, hydrophobic and self-cleaning properties [3,4,5,6,7,8,9,10].

In monumental architecture, the so-called patinas layers, consisting of calcium oxalate, calcium phosphate, and silica, constitute exemplar cases of well-preserved surfaces [11,12]. Inspired by our previous experience in analyzing these layers, among which are the patina layers from the Parthenon, the combination of these compounds within a consolidant product was one of the main objectives in the design of a colloidal dispersion capable of penetrating the stone and mortar surface [13,14,15]. This was achieved by designing a crack-free consolidant composite in a one-pot synthesis with great affinity to both carbonaceous and siliceous matrixes. Previous published work proved the performance of this consolidant (TCO) when applied to limestones and cementitious mortars [16].

Given that the consolidation efficiency of a product does not necessarily imply protective action, a different class of protective hybrid nanocomposites was designed in our lab, which provide simultaneously hydrophobic and self-cleaning properties on the lithoid substrates. These hybrid nanocomposites were designed to take advantage of the promising properties of TiO_2_ as a non-toxic photoactivated semiconductor exhibiting self-cleaning performance [17,18,19,20]. This research issued three nanocomposites (STP) synthesized at ambient conditions comprising TEOS, PDMS, TTIP, and oxalic acid dihydrate (Ox) [21,22,23]. The presence of the PDMS within the silica matrix reduced the surface energy of the xerogel, thus inducing hydrophobic properties to the treated surface [22,24]. The performance of these nanocomposites has been proven by extensive research and lab applications on their self-cleaning and hydrophobic properties, and finally by the significant role played by oxalic acid as a drying controller, chelating agent, peptizing agent, and hole-scavenger promoting photoactivity of TiO_2_. Moreover, photocatalytic performance can be highly enhanced when TiO_2_ exists in separate domains, as pointed out in our previous work [22,23].

Aiming at a holistic conservation approach and a user-friendly application procedure of synthesized nanocomposites, it deemed important to assess a three-layered treatment consisting of: (a) the consolidated structure with TCO; (b) the hydrophobic layer (SP) of TEOS-PDMS and (c) the self-cleaning layer (T) of TiO_2_ nanoparticles from titanium tetra-isopropoxide (TTIP) with oxalic acid (Ox) in the interface with environment. This strategy enabled TiO_2_ to reach a higher level of photoactivity, as it was located on the external layer, restricting its hydrophilicity and preventing the rest of the surface from being affected by the water. Despite the TiO_2_ hydrophilicity, the overall protection was not compromised due to the application of the second layer, totally dedicated to the shield of the substrate. 

In this paper, different lithoid substrates were consolidated by the TCO, aiming to provide a reaggregation of the lithoid units in-depth. Successively, the combined hydrophobic and self-cleaning layers will contribute to the protection and integrity of the surface. This adopted strategy first changed the already known hydrophilicity of TEOS by introducing PDMS, an agent providing hydrophobicity and crack-free gels [22,23,24,25], and, secondly, prevented TiO_2_ from penetrating stone and mortars, compromising photoactivation. In addition, the chemical compatibility between TEOS, PDMS, and TTIP enabled the anchorage of TiO_2_ in the silica matrix, preventing TiO_2_ from leaching. 

The performance and stability of the hydrophobic and self-cleaning (SP&T) nanocomposite was assessed by analytical techniques and ultraviolet (UV) ageing. The evaluation of the self-cleaning and hydrophobic performance was conducted on lithotypes with complementary properties, such as limestones with different porosity, a ceramic substrate, and two types of cementitious mortars, only one of which contained lime.

## 2. Materials and Method

### 2.1. Treated Substrates

The effectiveness of the consolidant TCO and both the hydrophobic (SP) and self-cleaning agents (T) was assessed on a total number of eighteen cylindrical samples with a diameter × height of 27 mm × 50 cm, 50 mm × 30 mm and 40 mm × 20 mm. The substrates can be grouped into three sub-categories according to their mineralogical composition:

- Limestones with different porosity, such as a porous biomicritic limestone called Alfas (AL), a travertine with highly compact structure (TR), and a calcitic sandstone (PRC) of high porosity and large pores;
- Ceramic material (CER) of compact structure;
- Two groups of cements mortars, designated as CLM (cement lime mortar) and CEM (cement mortar) with the binders of lime and cement in the CLM, in a ratio of Cement/Lime/Sand: 2/1/9; and cement only in CEM, in a ratio Cement/Sand: 1/3. The storage and curing of mortars followed the specifications described in the EN 1015-11 standard [26]. After demolding, the specimens were placed in a chamber at a temperature of 20 ± 2 °C and a relative humidity of 65 ± 5%.

The choice of the different lithotypes allowed the comparison of treatment performance in substrates with different composition and porosity.

The fresh specimens of all of the samples were first gently polished with the aid of an abrasive paper of fine grit size (P180 carborundum paper) and subsequently they were immersed into deionized water for 1.5 h with the purpose of removing the trapped salts and powder. Afterwards, the specimens were further rinsed with deionised water, dried in an oven at 80 °C for 3 days, then weighed and kept in a desiccator (WTB binder, Tuttlingen, Germany) vessel before the treatments.

### 2.2. Treatments with Consolidant (TCO) and Hydrophobic (SP)/Self-Cleaning (T) Agents

A consolidation treatment with TCO was performed on the different lithoid substrates in 2013 [16]. The treated samples were kept in laboratory conditions until now and the evaluation of their physicochemical and mechanical properties was carried out after three years of curing, in 2016, in order to assess the long-term efficiency of the consolidation in these stable conditions.

**Scheme 1:**
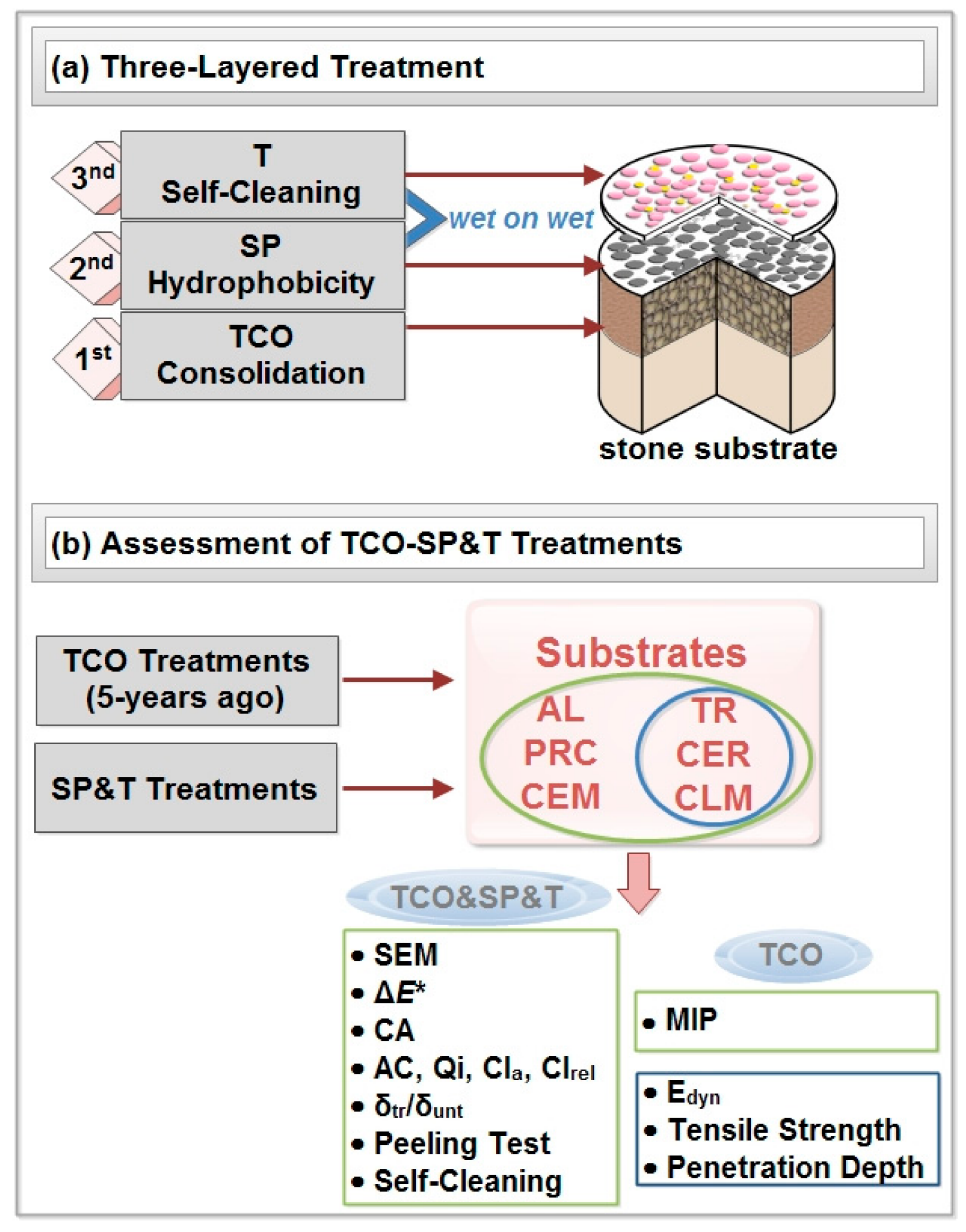
Schematic representation of the: (**a**) three-layered treatment and (**b**) techniques used for the evaluation of the treatment.

A protection treatment, with a different application protocol, followed by testing a simplified synthesis of the already studied STP [22]. The new protocol, illustrated in detail in Scheme 1a, consists of the separation of the hydrophobic phase (SP) of the nanocomposite from the self-cleaning phase (T). This was achieved by using tetraethyl orthosilicate (TEOS denoted as S, Sigma Aldrich, Darmstad, Germany) and hydroxyl-terminated polydimethylsiloxane (named as P, Sigma Aldrich, Darmstad, Germany) as main ingredients in the intermediate layer designated as SP, while the external layer consisted of TiO_2_ (T) from titanium (IV) isopropoxide (TTIP, Sigma Aldrich, Darmstad, Germany). In both SP and T, oxalic acid dihydrate (Ox, Panreac AppliChem, Barcelona, Spain) was used as a catalyst, chelating agent, and hole-scavenger. The final molar ratio of the precursors and solvents TEOS/EtOH/H_2_O/PDMS/TTIP was equal to 1/5.6/4/0.04/0.017, according to the synthesis of the SiO_2_-TiO_2_-PDMS STP-2 nanocomposite [22]. The result of the consequent application of the wet T layer on the wet surface pre-treated with SP layer will be denoted from now on as SP&T treatment. All the treatments, included TCO applications in 2013, were applied successively with the aid of a brush, as indicated in Scheme 1a.

### 2.3. Characterization of the Nanocomposites

The TCO nanocomposite synthesized in 2013 was fully characterized regarding its physicochemical, textural, and micro-structural properties and the obtained results are discussed in our previous scientific article in detail [16]. The TCO consolidant, in xerogel form, synthesized in 2013 remained under laboratory conditions up to 2017. Then it was microscopically assessed with the aid of a digital microscope USB Dino-Lite AM4515T5 Edge (AnMo Electronics Corporation, New Taipei, Taiwan) with a resolution of 1.3 megapixels and a zoom of 500×–550× using a color CMOS sensor and assisted by the software Dino Capture 2.0 (2017Q2 Copyright © 2017IDCP BV, AnMo Electronics Corporation, New Taipei, Taiwan). In order to study the nanostructure of the SP&T nanocomposite, SP and T dispersions were applied on a glass slide and analyzed using a Scanning Electron Microscopy (SEM, FEI Quanta Inspect D8334 instrument, Hillsboro, Oregon, United States,, operating at 25 kV) equipped with energy X-ray energy dispersive spectroscopy (X-EDS, Oxford Instruments 2000 ISIS, Oxford Instruments, Abingdon, Oxfordshire, UK) and Transmission Electron Microscopy (TEM, Jeol JEM 2010F, Jeol, Tokyo, Japan), equipped with X-ray energy dispersive spectroscopy (X-EDS) detector. For the TEM observations, SP and T dispersions applied on the glass slide were allowed until the solvent evaporated under ambient conditions. Afterwards, the film derived was smoothly grinded in an agate pestle and mortar and dispersed in high purity ethanol. A drop of the suspension was deposited onto a lacey carbon film supported on a 3 mm Cu grid and was allowed to evaporate under ambient conditions.

The chemical interaction and the effects of the UV ageing of the SP&T nanocomposite were evaluated through Fourier-Transform Infrared Spectroscopy (FTIR, Perkin-Elmer 1000 spectrometer, Perkin-Elmer, MA, USA) in the spectral range from 4000 to 400 cm^−1^, with spectral resolution of 4 cm^−1^ and 20 consecutive scans. The measured pellets derived from the powdering of xerogel samples and KBr (Sigma Aldrich, Sigma Aldrich, Darmstad, Germany, FTIR grade), in a weight ratio equal to 1:100.

Finally, the diffuse reflectance spectra (DRS) of the SP&T nanocomposite was measured using a Cary 5000 UV-Vis-NIR spectrophotometer (Varian, Palo Alto, CA, USA) in the range of 200–800 nm equipped with an integrating sphere DRA 2500. The Energy gap of the SP&T nanomaterials was calculated through the Tauc plots of (α·hv)^1/2^ versus E, allowing indirect transition, where α is the absorption coefficient (cm^−1^), h is the Planck’s constant (J.s), v is the light frequency (s^−1^) and E expresses the energy (eV) [27].

### 2.4. Assessment of the Consolidation and Protective Efficiency

An outline of the techniques applied for the assessing of the TCO and SP&T treatment is presented in Scheme 1b. After each layer of the TCO&SP&T treatment, the specimens were kept under laboratory conditions (RH = 60 ± 5%, T = 20 ± 2 °C) until a constant weight was achieved. The dry matter of the products absorbed was calculated, subtracting the weight before treatment from the constant weight of treated specimens. The microscopic appearance of the treated specimens was observed using the digital microscope USB Dino-Lite.

The aesthetic compatibility of the three-layered treatment was studied using a Konica Minolta, CM-2600d (Konica Minolta Optics, INS, Osaka, Japan), Vis spectrophotometer, adapted with a D65 illuminant at 8-degree viewing, in a wavelength range from 360 to 740 nm. The average values of the color parameters L*, α* and b*, derived from ten measurements of random areas of untreated and treated specimens used for the calculation of the total color difference expressed as ΔΕ* [21,28].

As far as the morphological structure of the substrates is concerned, both treated and untreated specimens, coated with a conducting layer of graphite, were observed by Scanning Electron Microscopy using a SEM Jeol JSM-5400 (Jeol, Tokyo, Japan) and FEI Quanta Inspect D8334 (Hillsboro, OR, USA).

Mercury Intrusion Porosimetry (MIP) using an Autoscan Porosimeter (Quantachrome Corporation, FL, USA), in the range from 0.005 to 200 μm, was used for the determination of both porosity and pore size distribution before and after consolidation treatment with TCO. The analyzed samples were previously kept at 70 °C for 180 min, aiming to remove the entrapped air and humidity in the pores.

The hydrophobic protection provided by the laboratory SP&T synthesized products was evaluated through the assessment of the water absorption by capillarity (WAC) according to the EN 15801:2010) [29] and the static contact angle (CA). Regarding the WAC, before and after treatment, the maximum water absorption per unit area (Qi, expressed in mg·cm^−1^) and the capillary water absorption coefficients (CA) in mg·cm^−1^·s^−1/2^ were calculated for all the substrates. Moreover, according to the literature [4,30] both the capillary index (CI) and the relative capillary index (CI_Rel_) were obtained from the WAC measurements. The CI was calculated through the formula: CI = $\int_{t0}^{\mathrm{tf}}\left(Q_{i}\right)\mathrm{dt}/Q_{\mathrm{tf}}\cdot t_{f}$, where $\int_{t0}^{\mathrm{tf}}\left(Q_{i}\right)\mathrm{dt}$ expresses the area under the absorption curve and the $Q_{\mathrm{tf}}$ denotes the total absorbed water per unit area at final time (t_f_). The CI_Rel_ further evaluates the effectiveness of the hydrophobic layer, as it compares the areas under the absorption curves observed from untreated and treated specimens. In particular, the CI_Rel_ was calculated by the equation: CI_Rel_ = $\int_{t0}^{\mathrm{tf}}f{\left(Q_{i}\right)}_{\mathrm{tr}}\mathrm{dt}/\int_{t0}^{\mathrm{tf}}f{\left(Q_{i}\right)}_{\mathrm{untr}}\mathrm{dt}$, where $\int_{t0}^{\mathrm{tf}}f{\left(Q_{i}\right)}_{\mathrm{tr}}\mathrm{dt}$ indicates the area under curve of the treated samples, whereas the $\int_{t0}^{\mathrm{tf}}f{\left(Q_{i}\right)}_{\mathrm{unt}}\mathrm{dt}$ expresses the corresponding area of the untreated specimens. As for the CA measurements, an optical tensionmeter (Thetalite TL 101, KSV, Biolin Scientific Attension, Gothenburg, Sweden) was used, determining the static contact angle (θs) of three treated and three untreated specimen surfaces according to EN Standard Procedure [31]. On different points of each specimen, several water droplets (~5 μL volume) were deposited close to the surfaces using a needle. Afterwards, the images of the droplets were further processed, revealing the average CA values for 0 and 20 s.

The water vapor permeability experiments were performed on cylindrical specimens of 40 mm × 20 mm (diameter × height) according to EN 15803:2010 [32]. The test was carried out before and after treatment, using a special home-made PVC apparatus. From the obtained results, the water vapor permeability δp expressed in kg·m^−2^·s^−1^·Pa^−1^, was calculated before the treatment (δp_untr_) and after the application of the materials (δp_tr_). The ratio of δp_tr_/δp_untr_ was calculated in order to assess the permeability of the substrates after the three layers were applied [4].

The estimation of the penetration depth and the stability of the TCO consolidant was based on the FTIR spectra from the analysis of powders originating from different depths of the treated surfaces. The consolidated specimens were cut in half and powders from different depths of the perpendicular surfaces were extracted with the aid of a dentist wheel. 

The mechanical properties used for the evaluation of the TCO effectiveness as a strengthening agent were: (i) the indirect tensile strength (Brazilian test) according to ASTM D3967-86 and (ii) the dynamic modulus of elasticity (E_dyn_) calculated from ultrasonic measurements according to EN 14579 (2004). The $E_{\mathrm{dyn}}$ was calculated through the equation: $E=d\cdot V^{2}$E, where d is the density of the sample and V the measured pulse velocity [16,28,33].

Furthermore, Vickers hardness of the ceramic substrate was calculated as the average value of the 6 measurements under loading of 300 g using a microhardness tester Future-Tech FM-700 (FUTURE-TECH CORP, Kawasaki-City, Japan).

The cohesion and adherence of the three-layered treatment were assessed by a peeling test according to the described methodology in the bibliography [34,35]. As a peeling means, a double-sided Tesa Powerbond Indoor (55740) adhesive tape (Global Headquarters—tesa SE, Norderstedt, Germany) was used.

### 2.5. Assessment of the Self-Cleaning Performance

The efficiency of the SP&T as self-cleaning layer for lithoid substrates was evaluated by monitoring the photo-degradation of stains deposited on the surfaces of specimens. In order to ensure both the homogeneous deposition and the rapid evaporation of the solvent, ethanolic solution 1 mM of Methylene Blue (MB, Panreac AppliChem, Barcelona, Spain) was used as the staining agent of the substrates [4]. 1 mL of the prepared MB solution was applied on each kind of specimen and the samples were kept in the dark for 42 h. Thereafter, all of the stained specimens, untreated and treated with SP&T, were subjected to a UV chamber equipped with four 8 W black light blue lamps, emitting in the near-UV range (315–400 nm, 3 mW·cm^−2^). The % Discoloration (%*D*) of the MB compound was calculated by monitoring the changes of the b* color parameter, using the Konica Minolta Vis-spectrophotometer, as previously described. The %D was obtained through the formula: $\%D=(\left|b_{\left(t\right)}^{*}-b_{\left(MB\right)}^{*}\right|/|b_{\left(MB\right)}^{*}-b_{\left(int\right)}^{*}|)*100$%*D* where $b_{\left(t\right)}^{*}$b is the value of the parameter after t hours of UV, $b_{\left(int\right)}^{*}$b*(int) represents the value of the coordinate $b^{*}$b* before the MB deposition and, finally, $b_{\left(MB\right)}^{*}$b*(MB) expresses the $b^{*}$b* value at time equal to 0 h [25]. Supporting evidence on the self-cleaning features of the stained specimens derived by capturing images under the optical Dino-Lite microscope (AnMo Electronics Corporation, New Taipei City, Taiwan).

Each assessment test for the product performance e.g., MIP, capillary water absorption, static contact angle, product penetration depth, spectrophotometric measurements, ultrasound velocity, indirect tensile strength, photocatalytic activity measurement, peeling test, referred to the average value of three samples per treatment.

## 3. Results and Discussion

### 3.1. Characterization of TCO and SP&T

**Figure 1:**
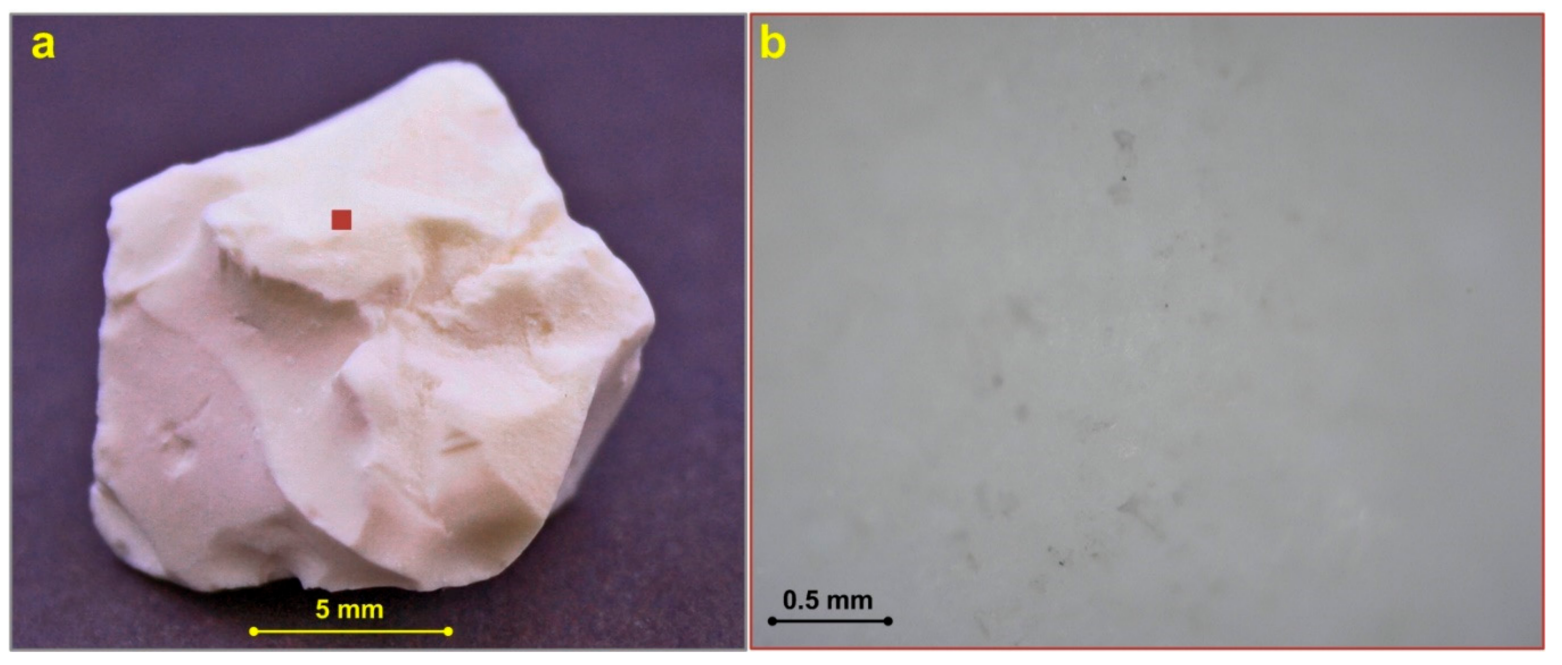
(**a**) Macroscopic and (**b**) optical microscopic images of the Treatments with Consolidant (TCO) consolidating agent after a five-year curing at ambient conditions.

The physicochemical, textural, and microstructural properties of TCO and the stability of the dispersion and xerogel, were discussed in detail in our previous work [16]. Macro- and micro-images of the hybrid nanocomposite after five years of curing, illustrated in Figure 1, evidenced that TCO maintained crack-free appearance and integrity over time. In particular, the microscopic appearance of the TCO xerogel depicted in Figure 1b, confirms that the nano-calcium oxalate was perfectly incorporated within the silica matrix, providing a dense and mesoporous microstructure.

Despite the detailed physicochemical, textural, and microstructural characterization of the STP nanocomposites in our previous works, it deemed important to further characterize the double-layered SP&T nanocomposite. Particular emphasis was placed on the characterization of the microstructure, the chemical interaction and stability obtained by SEM, TEM and FTIR analyses of the samples as prepared and aged UV.

**Figure 2:**
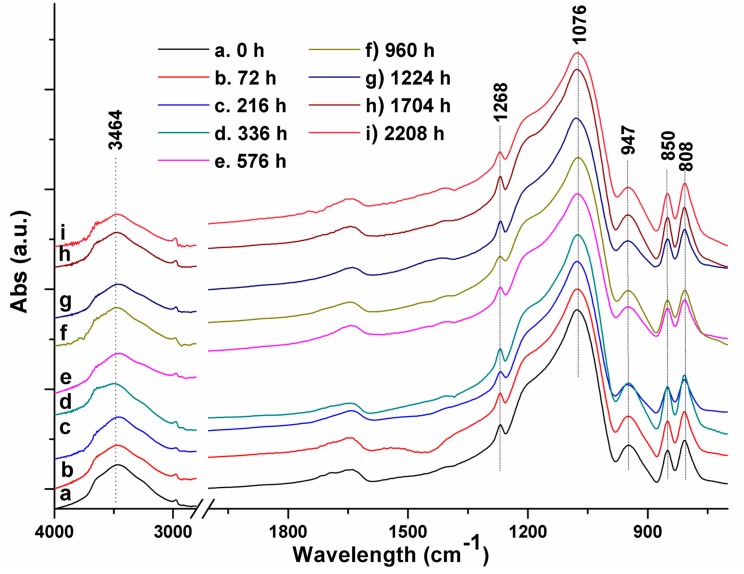
Fourier-Transform Infrared Spectroscopy (FTIR) analysis of the SP&T xerogel: (**a**) after the completion of the chemical reactions and (**b**–**i**) after the exposure to UV irradiation.

The chemical interaction, the bonds and the stability of the SP&T hybrid nanocomposite can be appreciated in the FTIR spectra of Figure 2, illustrating the SP and T dispersions separately and successively applied on a glass slide, when subjected to UV ageing at various time intervals. The chemical composition of the SP&T can be clearly seen in the spectrum (a) of Figure 2a, evidencing the hydrolysis of TEOS and the copolymerization between PDMS and silica matrix. The peaks located at 1076 and 808 cm^−1^ are attributed to the antisymmetric and symmetric stretching of the newly Si–O–Si bonds, respectively, indicating that hydrolysis and polymerization of TEOS occurred [22]. The peak observed at 850 cm^−1^ is directly assigned to the copolymerization of PDMS and TEOS [22,36,37]. The integration of the PDMS into the gel network is further confirmed both by the identification of the characteristic peak at 1268 cm^−1^ related to the symmetric bending of the –CH_3_ groups in the PDMS molecules [21], and the wide band in the spectral region of 3000–3700 cm^−1^ that is associated with the stretching of the O–H bonds of the hydroxyl terminated PDMS. The presence of the organic part of the PDMS is considered to be a crucial factor for the establishment of the hydrophobic behavior of the products. It is well-established that at the early stages of the TEOS, PDMS, and TTIP blend in sol phase, the overlapped silanol groups of PDMS, the unpolymerized parts of the silica matrix and the newly-formed Si–O–Ti bonds are denoted in the spectral region with a shoulder at ~960 cm^−1^ [21,36,37]. The shifting of this shoulder at ~947 cm^−1^ is the effect of the Si–O–Ti copolymerization [21,36]. Therefore, infrared spectra demonstrate the incorporation of the PDMS and TiO_2_ into the silica matrix, as it was indicated by the homogeneous structure in the microscoping images. 

The stability of the SP&T nanocomposite system was proven in the infrared spectra from (b) to (i) evidencing that copolymerization of TEOS, PDMS and TiO_2_ provided a stable nanocomposite not affected by the UV in a time interval of 2208 h. The peaks attributed to the Si–O–Si and Si–O–Ti bonds (at 1076 and 947 cm^−1^) after 2208 h of UV irradiation are at the same wavenumber as those of the unexposed material, evidenced the stability of the silica network. Moreover, PDMS peaks at 1268 and 850 cm^−1^ observed at the same wavenumbers in all of the spectra further corroborated the copolymerization of the SP&T nanocomposite.

**Figure 3:**
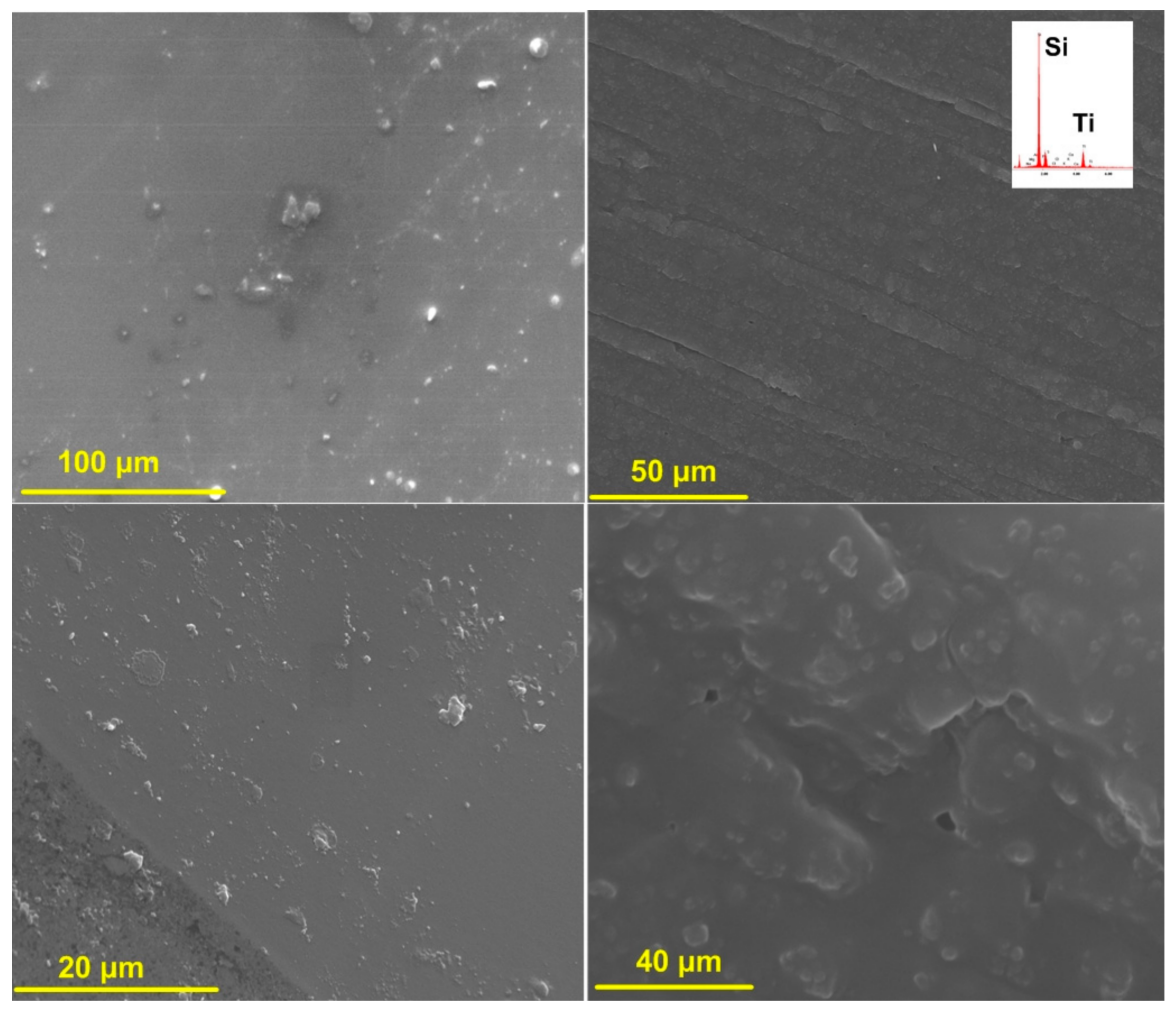
Scanning Electron Microscope (SEM) images of the SP&T xerogel obtained at different magnification.

A crack-free and homogeneous microstructure of the SP&T coatings can be appreciated in the SEM images of different magnification in Figure 3, further proving the copolymerization of SP and T coatings.

**Figure 4:**
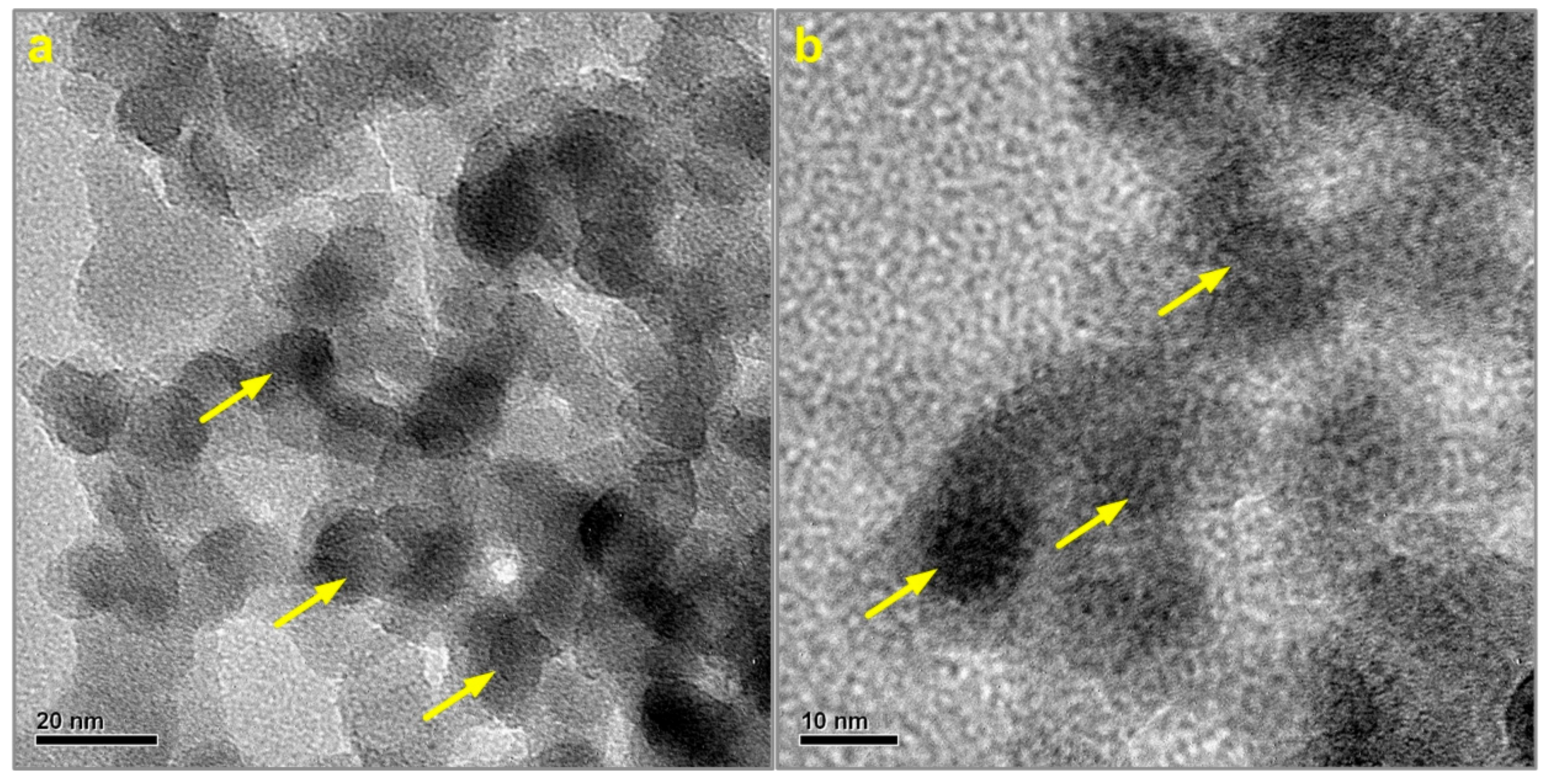
Transmission Electron Microscope (TEM) images of the SP&T nanocomposite indicating the amorphous phase of TiO_2_ nanoparticles and showing homogeneity (**a**) and interconnectivity (**b**).

The TEM images in bright-field mode of the SP&T nanocomposite, illustrated in Figure 4, confirmed the homogeneous structure of the nanocomposite in the nanoscale. An amorphous phase of TiO_2_ was observed to be fully incorporated into the silica and organic matrix of the nanocomposite. The TiO_2_ particles indicated with yellow arrows and corresponding to the darker part of the image, are well dispersed in the organosilane matrix, creating separate spherical domains of diameter from 5 to 10 nm [17,38]. Furthermore, the interconnection with the silica spherical domains of lighter color is clear in the images, thus pointing out the copolymerization observed in the FTIR spectra between silica and TiO_2_.

**Figure 5:**
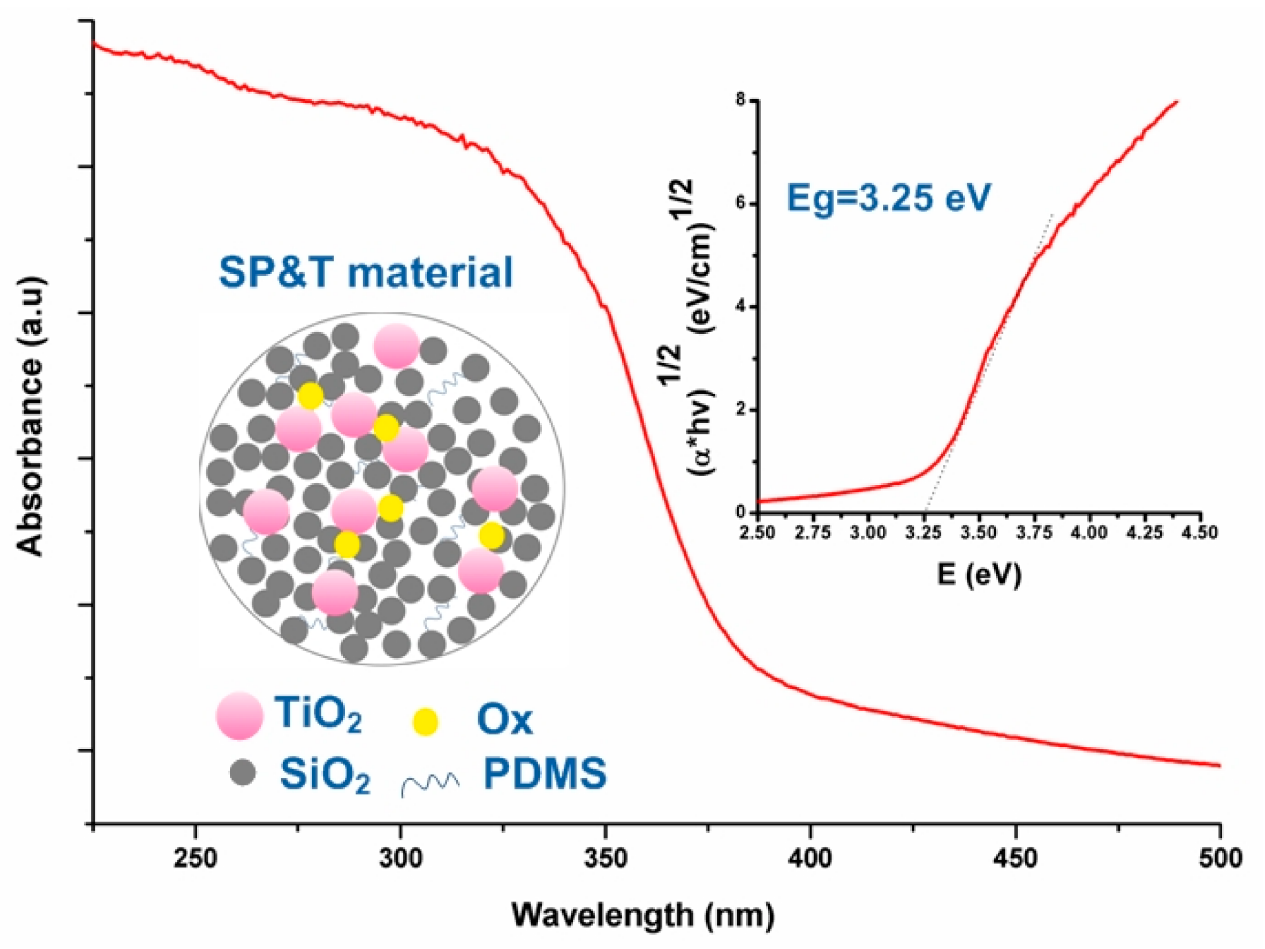
UV-Visible absorption spectrum and plot of (a*hv)^1/2^ versus Energy for the calculation of the band-gap energy (Eg) of SP&T material.

In the diffuse reflectance UV-Vis spectrum of SP&T in Figure 5, the recorded strong absorption edge located at 350–390 nm is clearly related to the photoactivity of T, given that SiO_2_ and organic matter did not absorb in this range of spectrum. In the inset plot of (a*hv)^1/2^ versus E for the band gap energy of SP&T nanocomposite, an energy gap (Eg) equal to 3.25 was calculated lower than the corresponding values calculated for the STP nanocomposites published in our previous research [22]. According to the literature, this value of Eg is consistent with previous measured Eg for amorphous TiO_2_ nanoparticles [39].

### 3.2. Evaluation of the Consolidating Performance of TCO 

#### 3.2.1. Microstructural Characteristics

The performance of TCO on alfas (AL), sandstone (PRC) and cement mortars (CEM) specimens has already been studied and presented in detail in our previous published work [16]. Therefore, in Table 1, listing the dry matter of TCO and the microstructural characteristics of the lythotypes, the values of the former referred to AL, PRC, and CEM specimens are not reported. Although the amount of TCO absorbed by the treated lithotypes can vary, a similar trend of TCO absorption was noticed. An exception to this trend is PRC, which needs ten times a higher amount of TCO to be saturated compared to the other specimens.

**Figure 6:**
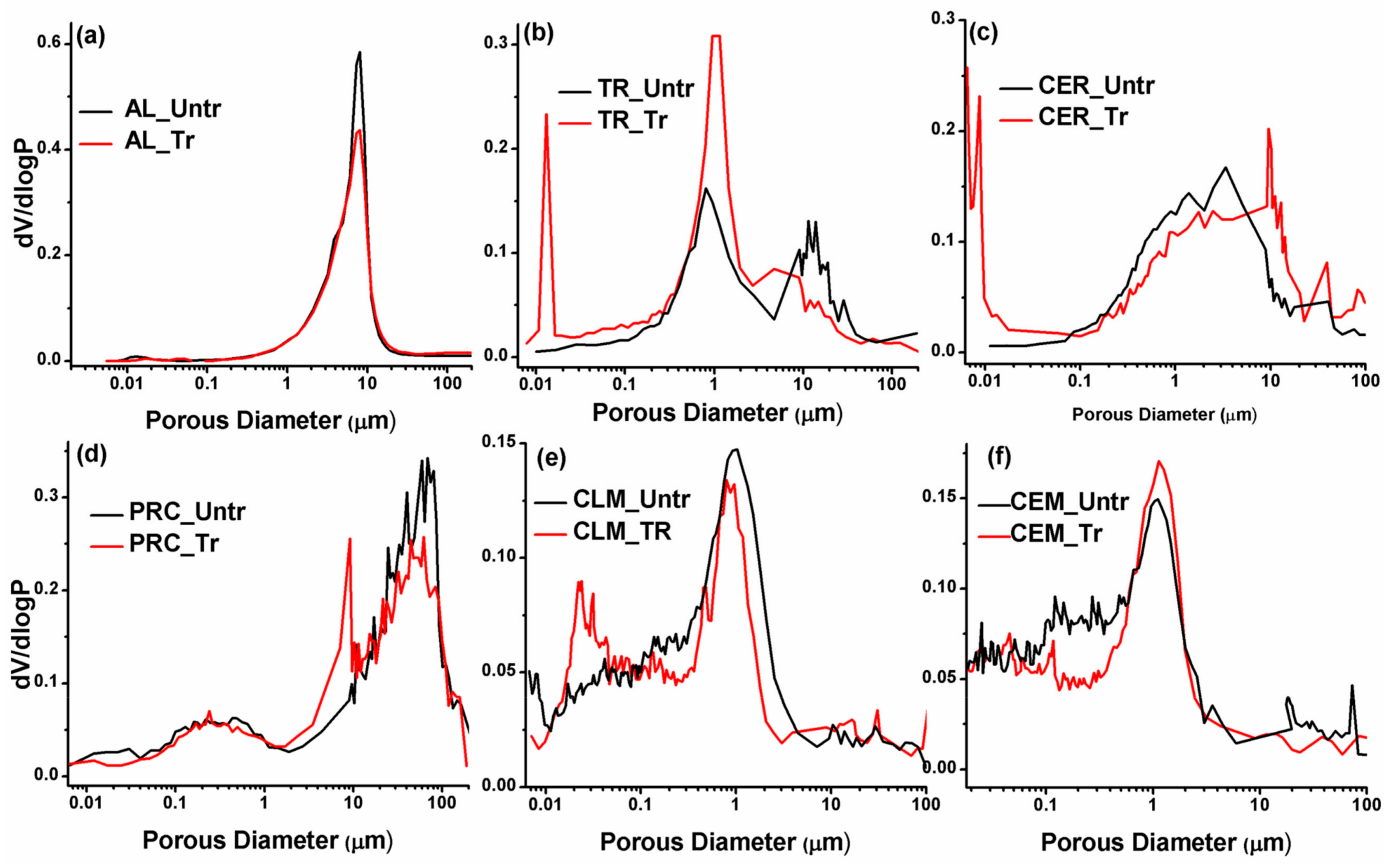
Representative graphs of pore size distribution, evaluated through Mercury Intrusion Porosimetry of: (**a**) AL; (**b**) TR; (**c**) CER; (**d**) PRC; (**e**) CLM and (**f**) CEM before and after consolidation with TCO.

The different dry matter of the treated lithoid substrates is in direct relationship with the values of the open porosity reported in Table 1 and the pore size distribution illustrated in Figure 6. The high amount of TCO absorbed by PRC specimens can be related to its high porosity (32.75%) and, more specifically, to its particular pore size distribution with large pores, which range from 5 to 100 μm, thus facilitating the impregnation with the consolidant. Notwithstanding the high porosity of AL (33.97%), it absorbed a lower amount of TCO (~40.00 mg/cm^2^ [16]), because of the narrow pores mainly distributed in the range of pore diameter 1–10 μm. Similarly to AL, the untreated TR, ceramic (CER), cement-lime mortars (CLM), and CEM absorbed relative low amounts of TCO, due to their low porosity and the prevalence of pores with diameter lower than 10 μm.

After treatment, the open porosity increased in travertime (TR) and CEM, decreased in AL and CER, and remained almost stable in CLM and PRC. The changes in the total surface area are not always in linear relationship with the changes in porosity. In TR travertine and CEM mortars the increase in porosity is also reflected in an increase of the total surface area, as a result of the newly-formed microporosity. The TCO microporous network can be observed in the pore size distribution of Figure 6, where new pores with a diameter lower than 0.1 μm appeared. Even though treated CER showed decreased porosity, newly-formed pores from TCO deposition on the pore walls, lower than 0.1 μm in diameter, led to a notable increase of total surface area (see also Table 1). On the other hand, in AL and PRC the decrease of total surface area is totally due to the partial occlusion of pores in the range of 5–50 μm. 

It is generally accepted that stones and mortars with pore sizes ranging from 0.1 to 10 μm are more vulnerable to salt and ice-induced damage [40,41]. However, in pore fractions below 0.1 μm a considerable degree of salt saturation is required in order for the solutions to penetrate and high stresses to arise from salt crystallization pressure [41]. Therefore, the pore size distribution of the material plays a decisive role in its resistance to salt and ice decay, as small pores are more vulnerable to salt and ice damage [41]. Pores in the range from 0.1 to 10 μm in diameter are generally related to poor performance against environmental salt-decay and the necessity to further protect the consolidated materials with a water repellent treatment [40]. 

#### 3.2.2. Mechanical Properties of the TR, CER, and CLM Treated Specimens

**Figure 7:**
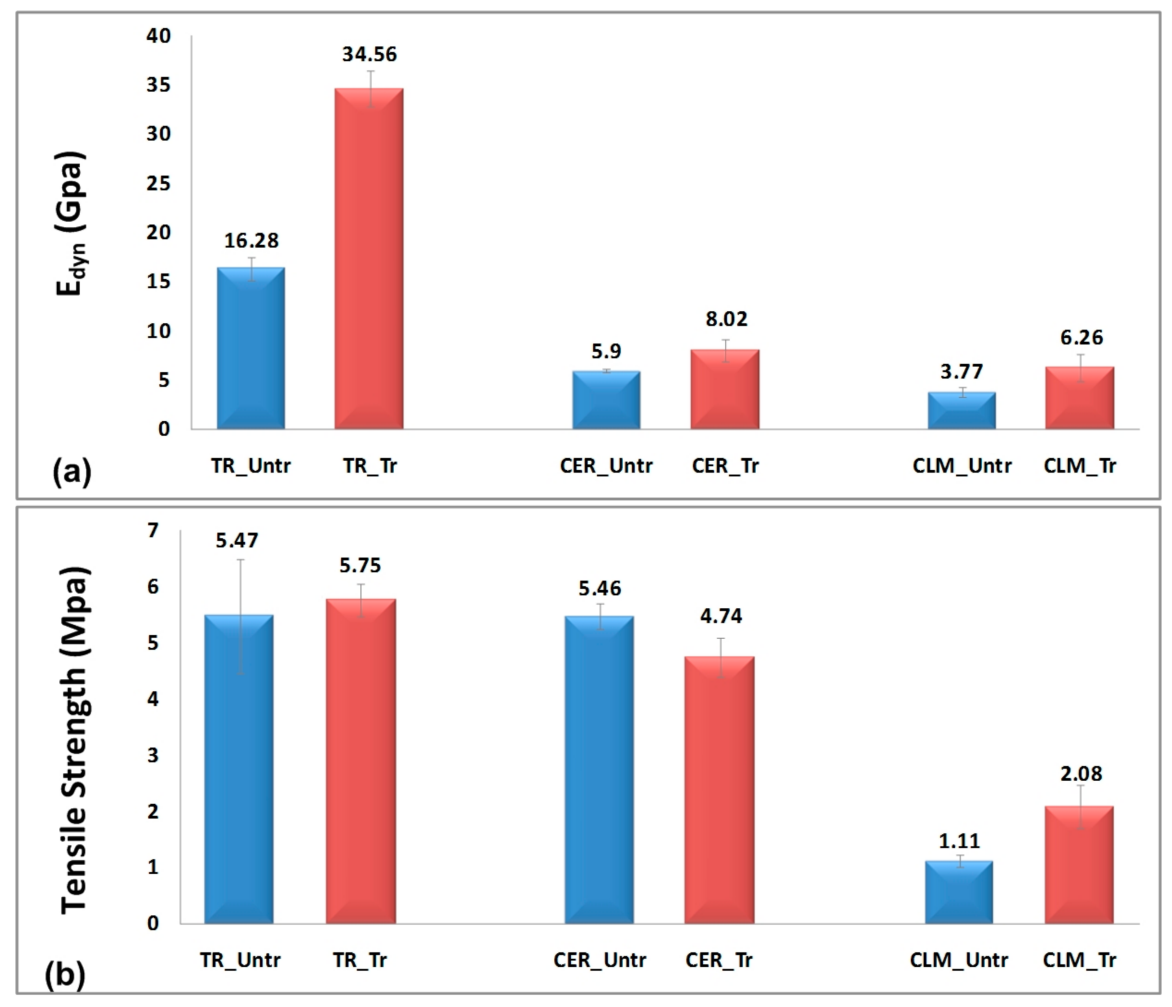
Mechanical Properties: (**a**) Dynamic modulus of elasticity (E_dyn_) and (**b**) Tensile Strength of TR, CER and CLM untreated and treated substrates.

The assessment of TCO as a strengthening agent was first evaluated by the dynamic modulus of elasticity (E_dyn_) derived from ultrasonic measurements before and after consolidation. All the treated specimens demonstrate a clear trend of E_dyn_ improvement due to the presence of TCO in their pore system, as detailed in Figure 7a, assuming that connectivity between the grains and consolidant was achieved, as it was also indicated by the increase of the total surface area in the MIP analysis.

Regarding the findings from the Brazilian indirect tensile test (Figure 7b), it can be concluded that TCO enhances the tensile resistance of both the TR and CLM, with increments of 5% and 87%, respectively. The almost double increase of the tensile resistance of CLM can be related to the great chemical affinity between cement-lime and TCO, since, the silica network conforms to the CSH components, and the calcite to the nano-calcium oxalate. The slight improvement of TR samples is most probably attributed to the already high values of their untreated counterparts. 

On the contrary, in the case of CER the resistance to tensile strength decreased by 13%, which does not seem to be in accordance with the increase of the E_dyn_ parameter values. By taking into account the Brazil values, it is reasonable to assume that after treatment the ceramic substrate became more fragile. In order to elucidate this discrepancy of the results, the CER substrate also underwent a Vickers micro-hardness test, which resulted in an increase of hardness by 42%. These findings are most probably due to the deposition of the TCO material in the first mm of the substrate, thus achieving a remarkable cohesion only of the grains close to the surface. This assumption can also be confirmed by the lowest penetration depth (4 mm) of TCO in ceramics compared to the other substrates, according to FTIR analysis (see Figure S1). To sum up, undesirable phenomena, such as over-strengthening that enable the formation of zones with different mechanical properties, are not evident in the treated specimens [42,43,44,45].

### 3.3. Assessment of the Hydrophobic and Self-Cleaning Properties of the SP&T Layers

The water repellency performance of TCO partially protected against harmful water action, as shown by previously measured values of the total water absorption by capillarity, due to the hydrophilic nature of the ingredients of TCO [13]. More specifically, in the formulation of TCO, hydrophilic components such as silica and calcium oxalate coexist. The calcium oxalate with lower solubility than calcite and provides an advantage to the TCO formulation against the disruptive effects of water action. The low contact angles of the treated TCO lithotypes explained in detail in the following paragraphs, support the need for additional treatment with protective agents. Therefore, the research addressed the issue with the application of two additional layers on the surfaces of the specimens treated with TCO. More specifically, the first layer is a hydrophobic product (SP), aiming to achieve the maximum protection of the building materials against water action, while the second one is a hydrophilic and photoactive material (T) providing self-cleaning properties to the surfaces. Table 2 reports the dry matter of those two layers applied on the surfaces already treated with TCO. PRC samples, as already registered for TCO, absorbed the highest amount of the product, confirming again the determining role of open porosity, since the treated with TCO PRC maintained high values of porosity similar to the untreated counterpart. AL, CER, and CEM samples absorbed similar quantities of the SP layer, ranging from 14.62 to 12.23 mg/cm^2^. The lowest dry matter was observed for the TR substrate, due to its low average pore size. As expected, the external T layer was absorbed in similar amounts by all the treated specimens, due to the already homogeneously distributed hydrophobic SP layer, which promoted successively the anchorage of TiO_2_ nanoparticles onto the silica matrix, thus preventing them from further penetration within the pores. The presence of the TiO_2_ nanoparticles in the interface with the atmosphere noticeably enhances their self-cleaning ability, as the active surface area of TiO_2_ became larger. 

#### 3.3.1. Assessment of the Aesthetic Compatibility and Surface Morphology after SP&T Treatment

The changes in color parameters induced by consolidation and protection treatments are summarized in Table 3. Taking into account that the generally accepted threshold value for ΔΕ* is equal to 5 [28], it can be inferred that the obtained values for the AL and TR limestones, the calcitic sandstone PRC, and the cementitious mortars CLM and CEM are close to the limit of acceptance adopted for treatments on monuments, which are generally characterized by higher restriction standards compared to the building sector. All the treated specimens, except for CER and PRC, became darker after treatment as expressed by the decrease in the L* color coordinate. The CER specimens became lighter from the deposition of the product on the surface, while the decrease of α* and b* are related to a prevalence of greenness and blueness of the treated specimens, respectively. The CLM and CEM treated mortars slightly surpass the acceptability limit and their color differences are related to a blueness in CLM and to a darkness in CEM.

Figures S2–S7 illustrate the optical microscopic images derived after treatment with SP&T. The SP&T layers seem to be in harmony with the substrates without inducing any modifications on the surfaces. However, in the case of CER, the ΔE* values exceeded the acceptability limits, establishing an aesthetic incompatibility of the TCO&SP&T treatment.

The surface morphological features, as observed in the SEM images of Figure S8, comparing untreated and treated specimens with the three-layered nanocomposites, are relative to the peculiar characteristics of the substrates. The untreated surface images remarkably differed in microstructure, crystalline morphology, and surface area, ranging from the porous and high total surface area of PRC, to the less porous and more compact AL, TR, and CLM, and finally to the massive microstructure of CER and CEM. In the treated specimens (S8, a_2_–f_2_), the TCO&SP&T layered treatment seems to preserve to a great extent the surface features by covering the original crystal network and developing a microporous network on the surface pattern. The treated stones and ceramic surfaces exhibit areas with similar morphological features reminding the homogenization of the SP&T layer when observed under SEM (see Figure 3). Likewise, the treated surfaces of mortars show more homogeneous features owing to similarities in matrix and treatment.

#### 3.3.2. Assessment of the Water Repellency of SP&T

Table 4 compares the static contact angle (CA) measured in 0 and 20 s for untreated, the treated lithotypes first with TCO and then with SP&T. First it should be highlighted that in the untreated AL, PRC, and CEM the surface hydrophilicity led to the absorption of the water drops, which resulted in the measurement not being performed, even in 0 s. In the TR and CER specimens treated with TCO hydrophobicity slightly improved, as evidenced by the CAs obtained in 0 s, mainly due to the increase of the total surface area (see also Table 1). The noticeable increase in CA achieved with SP&T is attributed to the inherent hydrophobicity of the PDMS chains incorporated into the silica matrix [22,24,46,47]. The peeling test results listed in Table 5, offer compelling evidence of the degree of the consolidation effectiveness of TCO and the adhesion with the SP&T coatings. After treatment with TCO&SP&T the specimens exhibited a noticeable reduction of the material removed by means of adhesive tape. This high reduction of the material removed, starting from 62% in CLM and ranging from 91–98% in all the other treated specimens, evidently, pointed out the reaggregation of the grains and the adhesion of the protective layers on the substrates.

The surface wettability data, before and after the three-layered treatment, at longer time intervals expressed by the Capillary Water Absorption Coefficient (AC), Maximum Water Absorbed per unit area (Qi), Absolute Capillary Index (CΙ) and Relative Capillary Index (CΙ_Rel_) are reported in Table 6. The surface high wettability in short time expressed by either low or not perceivable CA values, was maintained at the capillary water absorption test with high values of the established coefficients AC, Qi, and CI. Treatment induced a remarkable reduction in the water absorbed, ranging from 74% for CEM to 97% for CER. Comparing the values of the relative capillary index (CI_Rel_), AL and CER were classified as the least wetted surfaces with values of 0.04 and 0.02, respectively, followed by TR with 0.11, mortars with 0.16, and PRC with 0.30. 

The differentiation of the reduction in water absorption of the treated specimens can be explained by the changes induced in porosity and pore size distribution by the TCO treatment along with the maintenance of the surface pattern after SP&T as observed in SEM images.

An important requirement of protective treatments is the ability of the applied layers not to obstruct the water vapor transmission through the treated surface. The δ_t_/δ_unt_ values of the water vapor permeability, comparing treated to untreated specimens, revealed that the three-layered proposed holistic conservation treatment decreased the surface transpiration without exceeding the acceptability limits [40,48]. Although, the treated PRC reduced the water vapor permeability by 74%, all the other treated specimens exhibited values, which guarantee that moisture will not be entrapped in the material.

#### 3.3.3. Effectiveness of the Self-Cleaning Efficiency of SP&T

**Figure 8:**
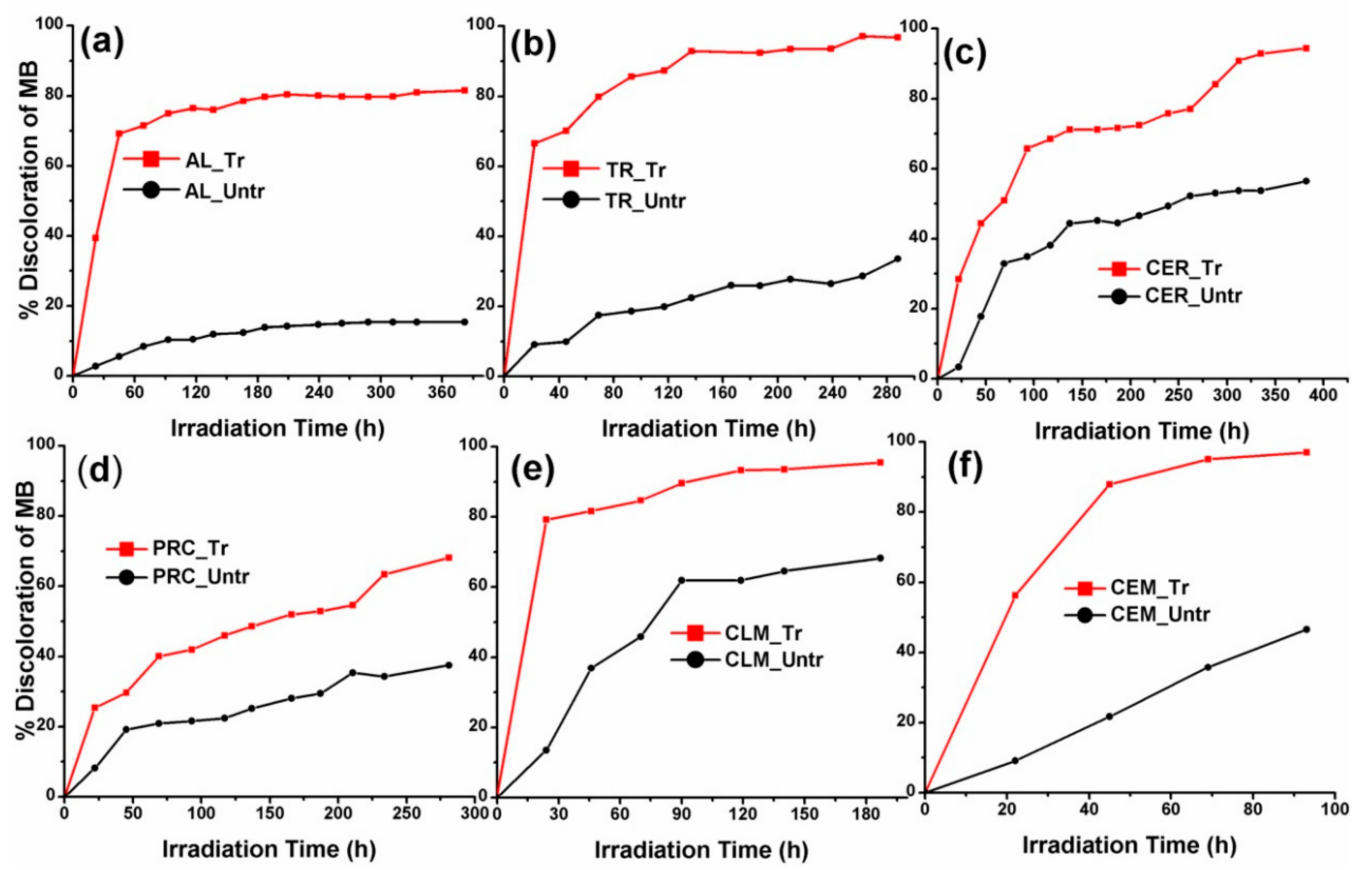
Assessment of the self-cleaning efficiency of the SP&T: (**a**) AL; (**b**) Tr; (**c**) CER; (**d**) PRC; (**e**) CLM and (**f**) CEM treated substrates.

Interesting results were obtained by the ability of the SP&T coating to discolor the stains of methylene blue (MB) after exposure to UV irradiation (Figure 8). The plots indicated that surface composition played a determining role in the discoloration of MB. This is a rational assumption, since the absorbed amount of T differed insignificantly in the treated lithotypes, except for TR, which absorbed half the amount of T compared to the others. The discoloration ranges from 70% for PRC to 80–100% for the other lithotypes. The MB stains were completely discolorated (D equal to 100%) in cement mortars CLM and CEM at the shortest time, as those mortars contain cement and lime. The latter fairly contributed to the decomposition of the organic MB, as pointed out in the plot of the untreated CLM, which also reached a 60% of MB discoloration in 90 h. Good photocatalytic performance was registered for limestones AL and TR, which reached a plateau at about 150 h of irradiation, with discoloration of 80% and 90%, respectively. The good photocatalytic performance of TR can be correlated to the highest CA measured, implying that the TiO_2_ nanoparticles, forming a “lotus effect” on the T layer through the Si–O–Ti bonds, photoactivated to a higher degree. It is well established that highly dispersed phases contribute to the avoidance of TiO_2_ aggregates and facilitate photoactivity [49]. Despite the amorphous TiO_2_ in the three-layered treatment, the SiO_2_ matrix functioned as a means of absorption, increasing the dispersion of nano-TiO_2_ and leading to the Si–O–Ti hetero-linkages upon the wet-on-wet SP&T application [37,50]. Essentially, the dispersion of the TiO_2_ nanoparticles within the silica matrix were evidenced in the TEM images previously reported (Figure 4). Moreover, the TiO_2_ immobilization onto construction materials may be also exploited in the wastewater treatments for the removal of biopersistent pollutants, as evidenced in the photodegradation of MB under UV light with commercial anatase deposited onto a cementitious channel [51,52]. 

The images of the stained-untreated and -treated specimens subjected to UV irradiation and obtained under the optical microscope are illustrated in Figures S2–S7. In those images the self-cleaning performance of the SP&T treatment previously described can be appreciated.

By correlating the above obtained results of the photocatalytic activity of the SP&T with the copolymerization of silica network and PDMS, along with the interaction with nano-TiO_2_, as evidenced by the Si–O–Ti bonds in the FTIR data, the compatibility of the proposed three-layered treatment emerged. The photoactivation experiments demonstrated that the three-layered treatment, on different substrates in terms of microstructure and composition, functioned as a means of adhesion of the TiO_2_ nanoparticles incorporated into the silica network, thus preventing them from penetrating the porous matrix and from leaching. Therefore, the required compatibility of nanocomposites tailored to a holistic conservation approach of monumental facades has been thoroughly studied in this research and both advantages and drawbacks were carefully taken into consideration.

## 4. Conclusions

The demand for ecological and minimal energetic solutions imposes specific conservation and maintenance strategies towards the amelioration of strengthening, hydrophobic, and self-cleaning properties of the construction materials of monuments. To fulfil these objectives this research proposed and evaluated a three-layered nanostructured treatment consisting of: (a) a TEOS-nano-calcium oxalate nanocomposite (TCO) with strengthening properties, exhibiting affinity to carbonate and siliceous substrates; (b) a hydrophobic TEOS-PDMS (SP) nanocomposite and (c) nano-TiO_2_ from a TTIP and oxalic acid dispersion (T), with self-cleaning properties, due to nano-TiO_2_ incorporation into the SP layer in the interface with atmosphere, enhancing photoactivation. It is worth highlighting that in this research a combination of the three-layered compatible nanocomposites was attempted for the first time, proposing also a different application protocol for the PDMS-SiO_2_-TiO_2_ nanocomposite, targeted to enhance photocatalysis. This nanostructured SP&T nanocomposite was resistant to UV ageing and nano-TiO_2_ was also incorporated into the silica matrix, as proven by FTIR, SEM, TEM, and optical microscopy analyses. Furthermore, the SP&T incorporation did not induce any modification in the values of Eg for the nano-TiO_2_, indicating that the photocatalytic properties of nano-TiO_2_ were maintained. The treated lithotypes and mortars with TCO showed strengthening properties along with a porosity and pore size distribution insignificantly modified. However, in the case of travertine and ceramic, the newly-formed microporous structure may result in mediocre resistance in conditions of salt crystallization and freezing-thawing cycles. In the ceramic substrate the low penetration depth of TCO was also reflected by a slight decrease of the tensile strength. The cement mortars performed well after the application of the three-layered treatment. In the framework of a Horizon 2020 project “InnovaConcrete” currently in progress, aiming to preserve concrete-based monuments, TCO is one of the studied impregnation nanocomposites to restore the properties of damaged concrete.

The hydrophobic properties improved after SP&T treatment for all the studied substrates, as proven by the contact angle and capillary water absorption values. The three-layered treatment reaggregated the structure and adhered well to the surface, since an insignificant amount of material was removed with the peeling test. The water vapor permeability is ensured for the majority of the studied lithotypes with an exception of PRC, where the significant reduction is related to the high amount of TCO&SP&T absorbed. The aesthetic compatibility of the TCO&SP&T treatment was compromised in the case of ceramic material with values exceeding the acceptability limits. 

Discolouration experiments of MB on the various lithotypes demonstrated that the new application protocol of TCO&SP&T treatment exhibited enhanced self-cleaning performance, achieving total discoloration of MB in the majority of the substrates as a result of the incorporation of nano-TiO_2_ into the silica network, as evidenced by the formation of stable Si–O–Ti bonds identified by FTIR.

The TCO&SP&T three-layered treatment ensured the reaggregation of the grains in the majority of studied lithotypes with different petrographic characteristics and porosity. Moreover, protection against water action with the SP hydrophobic layer and a self-cleaning ability with the nano-TiO_2_ anchored on the interface of the protective layer/atmosphere, were also accomplished.

## Figures and Tables

**Table 1:** Dry matter (mg/cm^2^) of the xerogel, % Porosity, Total Surface Area (m^2^/g) and Average Pore Diameter (μm) of untreated and treated substrates with TCO.

Property	AL: Alfas		TR: Travertine		CER: Ceramic		PRC: Sandstone		CLM: Cement-Lime Mortars		CEM: Cement Mortars	
	Untr	Tr_TCO	Untr	Tr_TCO	Unt	Tr_TCO	Unt	Tr_TCO	Unt	Tr_TCO	Unt	Tr_TCO
Dry matter (mg/cm^2^)	-	- ^1^	-	21.87 (±8.59)	-	25.51 (±1.61)	-	- ^1^	-	33.02 (±2.24)	-	- ^1^
% Porosity (MIP)	33.97	28.05	18.01	23.94	22.73	20.14	32.75	32.69	20.34	20.8	13.33	19.38
Total Surface Area (MIP) (m^2^/g)	1.21	0.50	0.99	1.98	1.06	6.46	2.54	1.86	4.82	4.86	0.34	4.14
Average Pore Diameter (MIP) (μm)	0.711	1.247	0.334	0.187	0.428	0.067	0.363	0.407	0.081	0.079	0.77	0.097

^1^ Already Published Results [16], MIP: Mercury Intrusion Porosimetry.

**Table 2:** Dry matter (mg/cm^2^) of the SP and T layers absorbed by the substrates.

Substrate	Dry Matter (mg/cm^2^)	
	Tr_SP	Tr_T
AL	14.62 (±2.1)	0.63 (±0.1)
TR	5.60 (±0.33)	0.25 (±0.01)
CER	12.23 (±2.3)	0.50 (±0.01)
PRC	22.0 (±3.1)	0.51 (±0.02)
CLM	8.15 (±1.10)	0.51 (±0.01)
CEM	12.23 (±1.8)	0.51 (±0.02)

**Table 3:** Color changes of L*, α* and b* parameters before and after treatment with TCO&SP&T layers.

Substrate	Color Parameters			
	ΔL*	Δα*	Δb*	ΔΕ*
AL	−4.55 (±0.45)	0.14 (±0.03)	0.14 (±0.07)	4.55 (±1.06)
TR	−1.91 (±0.61)	−1.48 (±0.19)	−4.04 (±0.18)	4.71 (±0.96)
CER	6.19 (±0.96)	−7.15 (±0.87)	−11.38 (±0.89)	14.85 (±1.36)
PRC	0.86 (±0.77)	0.75 (±0.10)	−1.02 (±0.17)	1.53 (±0.54)
CLM	−1.02 (±0.39)	−0.83 (±0.31)	−6.43 (±0.11)	6.57 (±1.01)
CEM	−7.82 (±0.92)	−0.09 (±0.01)	−1.34 (±0.27)	7.94 (±1.02)

**Table 4:** Contact Angle (θ°) measurements of untreated and three-layered treated surfaces.

Substrate	Time (s)	CA (θ°)		
		Untr	Tr_TCO	Tr_SP&T
AL	0	-	40 (±5)	107 (±7)
	20	-	-	99 (±7)
TR	0	84 (±9)	98 (±3)	126 (±9)
	20	41 (±12)	60 (±22)	121 (±10)
CER	0	34 (±11)	82 (±9)	112 (±9)
	20	-	42 (±15)	106 (±7)
PRC	0	-	-	111 (±7)
	20	-	-	110 (±6)
CLM	0	40 (±7)	38 (±11)	95 (±8)
	20	-	-	82 (±8)
CEM	0	-	30 (±6)	110 (±13)
	20	-	-	102 (±12)

- Not detected.

**Table 5:** Results obtained through the performance of peeling test.

Substrate		Total Amount (mg)	% Reduction of the Removal Substrate after the Treatment
AL	Untr	0.37 (±0.01)	−95
	Tr_TCO&SP&T	0.02 (±0.00)	
TR	Untr	2.00 (±0.05)	−91
	Tr_TCO&SP&T	0.18 (±0.01)	
CER	Untr	1.03 (±0.04)	−91
	Tr_TCO&SP&T	0.09 (±0.01)	
PRC	Untr	6.59 (±0.6)	−94
	Tr_TCO&SP&T	0.42 (±0.04)	
CLM	Untr	2.85 (±0.5)	−62
	Tr_TCO&SP&T	1.09 (±0.01)	
CEM	Untr	1.54 (±0.1)	−98
	Tr_TCO&SP&T	0.03 (±0.00)	

**Table 6:** Capillary Water Absorption Coefficient (AC, mg·cm^−2^·s^−1/2^), Maximum Water Absorbed per unit area (Qi, mg·cm^−2^), Absolute Capillary Index (CΙ) and Relative capillary index (CΙ_Rel_) and δ_t_/δ_unt_ values of the water vapor permeability of untreated and treated substrates.

Property	AL		TR		CER		PRC		CLM		CEM	
	Untr	Tr_SP&T	Untr	Tr_SP&T	Unt	Tr_SP&T	Unt	Tr_SP&T	Unt	Tr_SP&T	Unt	Tr_SP&T
AC (mg·cm^−2^·s^−1/2^)	22.11 (±6.57)	0.15 (±0.01)	1.09 (±0.01)	0.16 (±0.01)	10.64 (±0.41)	0.12 (±0.01)	33.82 (±0.97)	4.77 (±0.43)	18.06 (±0.68)	1.05(±0.29)	12.35 (±2.04)	0.84(±0.12)
Qi (mg·cm^−2^)	1250.96 (±32.43)	172.23 (±9.30)	191.08 (±16.21)	21.91 (±6.21)	991.93 (±28.17)	34.65 (±2.70)	1982.68 (±56.60)	801.68 (±21.70)	1040.34 (±36.86)	334.47 (±22.44)	1014.86 (±21.67)	265.46 (±5.80)
CΙ	0.90 (±0.01)	0.24 (±0.01)	0.72 (±0.02)	0.40 (±0.01)	0.86 (±0.02)	0.46 (±0.02)	0.89 (±0.01)	0.65 (±0.01)	0.90 (±0.00)	0.47 (±0.029)	0.87 (±0.01)	0.38 (±0.03)
CΙ_Rel_	0.04 (±0.00)		0.11 (±0.02)		0.02 (±0.01)		0.30 (±0.04)		0.17 (±0.01)		0.16 (±0.01)	
δp_tr_/δp_unt_	0.67 (±0.08)		0.70 (±0.10)		0.75 (±0.07)		0.26 (±0.02)		0.60 (±0.07)		0.50 (±0.03)	

## Supplementary Materials

The following are available online at http://www.mdpi.com/1996-1944/11/5/684/s1, Figure S1: FTIR spectra indicating the penetration depth of TCO in: (a) TR, (b) CER and (c) CLM substrate. Figure S2: Optical Microscopy images of AL substrate: untreated (a), treated with TCO&SP&T (b); treated and stained: before (c) and after (d) UV irradiation; untreated and stained: before (e) and after (f) UV irradiation. Figure S3: Optical Microscopy images of TR substrate: untreated (a), treated with TCO&SP&T (b); treated and stained: before (c) and after (d) UV irradiation; untreated and stained: before (e) and after (f) UV irradiation. Figure S4: Optical Microscopy images of CER substrate: untreated (a), treated with TCO&SP&T (b); treated and stained: before (c) and after (d) UV irradiation; untreated and stained: before (e) and after (f) UV irradiation. Figure S5: Optical Microscopy images of TR substrate: untreated (a), treated with TCO&SP&T (b); treated and stained: before (c) and after (d) UV irradiation; untreated and stained: before (e) and after (f) UV irradiation. Figure S6: Optical Microscopy images of CLM substrate: untreated (a), treated with TCO&SP&T (b); treated and stained: before (c) and after (d) UV irradiation; untreated and stained: before (e) and after (f) UV irradiation. Figure S7: Optical Microscopy images of CEM substrate: untreated (a), treated with TCO&SP&T (b); treated and stained: before (c) and after (d) UV irradiation; untreated and stained: before (e) and after (f) UV irradiation. Figure S8: SEM images of untreated (a_1_–f_1_) and treated with TCO&SP&T (a_2_–f_2_) for AL, TR, CER, PRC, CLM, CEM substrates, respectively.
