# Supplementary material for: Conservation of Monuments by a Three-Layered Compatible Treatment of TEOS-Nano-Calcium Oxalate Consolidant and TEOS-PDMS-TiO2 Hydrophobic/Photoactive Hybrid Nanomaterials
Source: Materials (Basel). 2018 Apr 27;11(5):684. doi: 10.3390/ma11050684 (PMC5978061; doi:10.3390/ma11050684)
Supplement: Supplementary file 1 [file materials-11-00684-s001.pdf]

Supplementary

Conservation of Monuments by a Three-layered Compatible Treatment of TEOS-nano-Calcium Oxalate Consolidant and TEOS-PDMS-TiO_2 Hydrophobic/Photoactive Hybrid Nanomaterials

Chrysi Kapridaki, Anastasia Verganelaki, Pipina Dimitriadou and Pagona Maravelaki-Kalaitzaki*

Supplementary Materials

S1. Evaluation of the TCO Penetration depth

Powders originating from the surface layer of the substrates and from different depths were examined in order to detect the maximum depth of the TCO presence. In Figure S1, representative FTIR spectra of the treated substrates are illustrated showing the penetration depth of the TCO material after the consolidation process. The two prevailing peaks of calcium oxalate, which appeared at 1622, 1320 cm^{-1}, were mainly monitored in order to evaluate the existence of calcium oxalate. In TR travertine, the TCO product penetrated up to a depth of 20 mm from the surface, whereas in the cementitious lime materials (CLM) TCO was measured at 15 mm. Regarding CER, the spectra revealed the presence of TCO at 4 mm. The variations of the penetration depth of TCO are probably ascribed to the different porous system of the substrates.

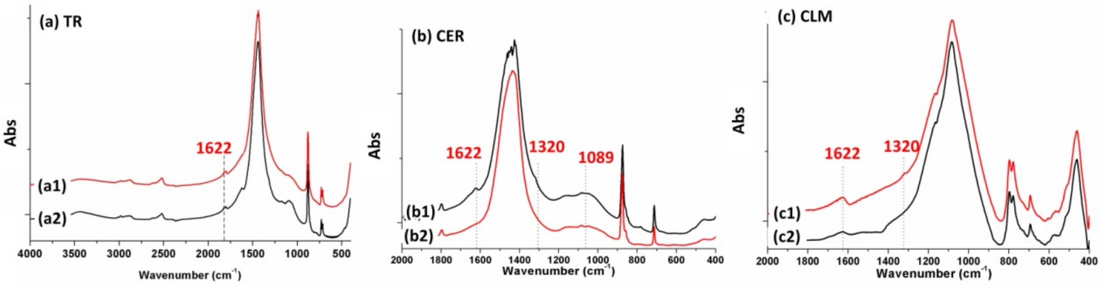

Figure S1. FTIR spectra indicating the penetration depth of TCO in: (a) TR at 0 mm (a1) and 20 mm (a2) from the surface, (b) CER at 0 mm (b1) and 4 mm (b2) from the surface and (c) CLM at 0 mm (c1) and 15 mm (c 2) from the surface.

S2. Optical Microscopy Images of Untreated and Treated Substrates

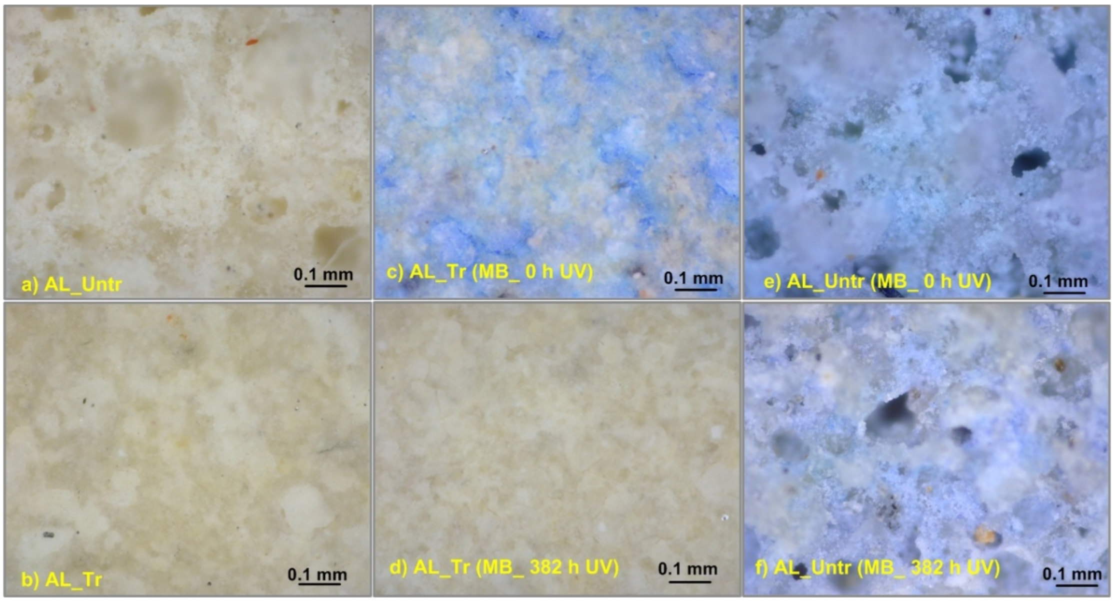

Figure S2. Optical Microscopy images of AL substrate: untreated (a), treated with TCO&SP&T (b); treated and stained: before (c) and after (d) UV irradiation; untreated and stained: before (e) and after (f) UV irradiation.

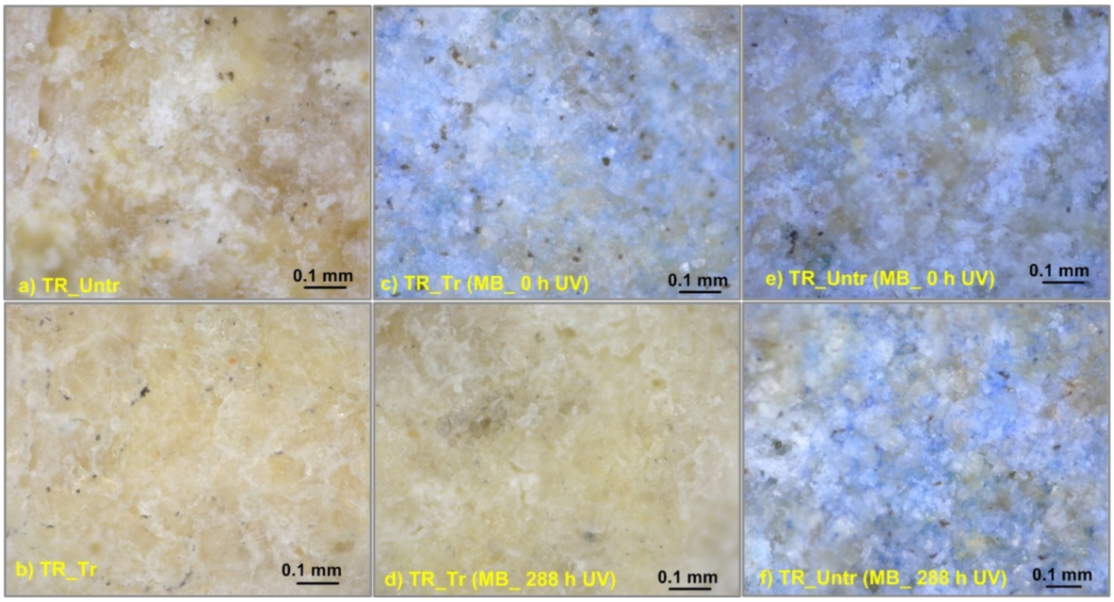

Figure S3. Optical Microscopy images of TR substrate: untreated (a), treated with TCO&SP&T (b); treated and stained: before (c) and after (d) UV irradiation; untreated and stained: before (e) and after (f) UV irradiation.

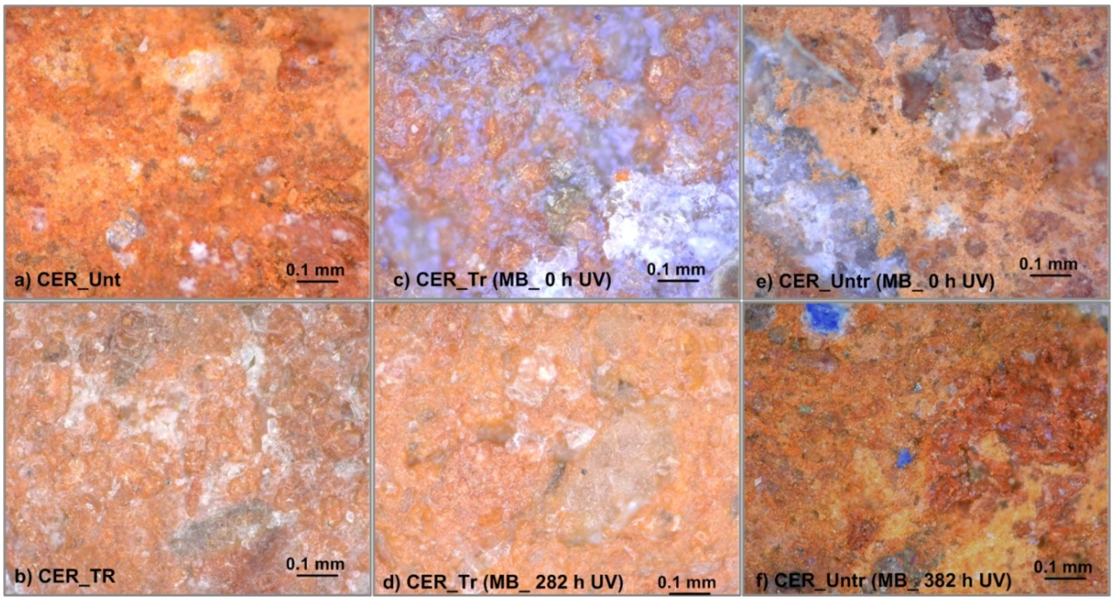

Figure S4. Optical Microscopy images of CER substrate: untreated (a), treated with TCO&SP&T (b); treated and stained: before (c) and after (d) UV irradiation; untreated and stained: before (e) and after (f) UV irradiation.

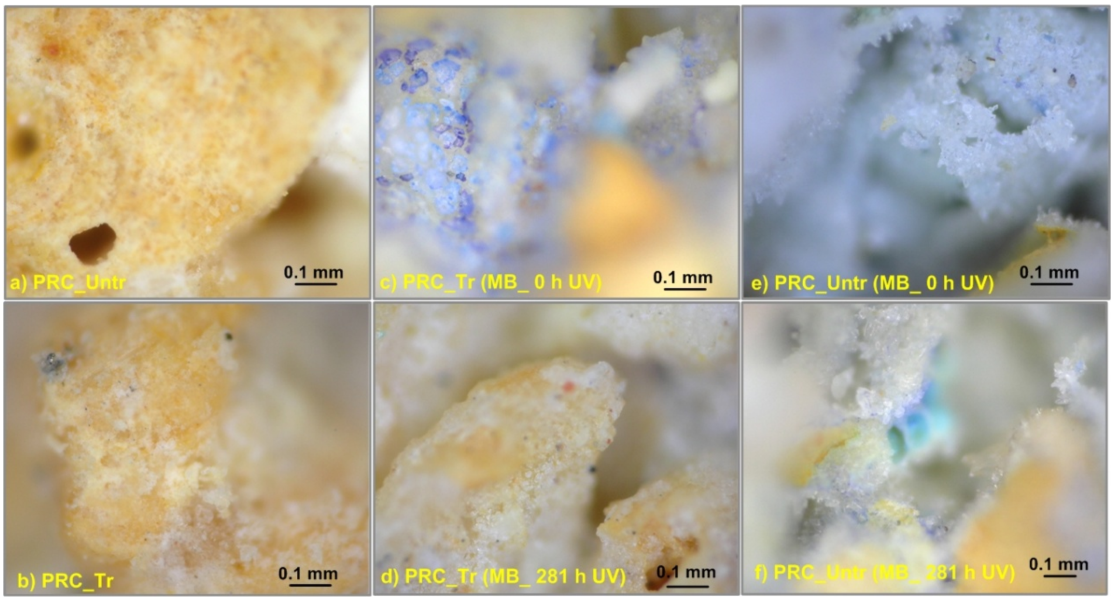

Figure S5. Optical Microscopy images of TR substrate: untreated (a), treated with TCO&SP&T (b); treated and stained: before (c) and after (d) UV irradiation; untreated and stained: before (e) and after (f) UV irradiation.

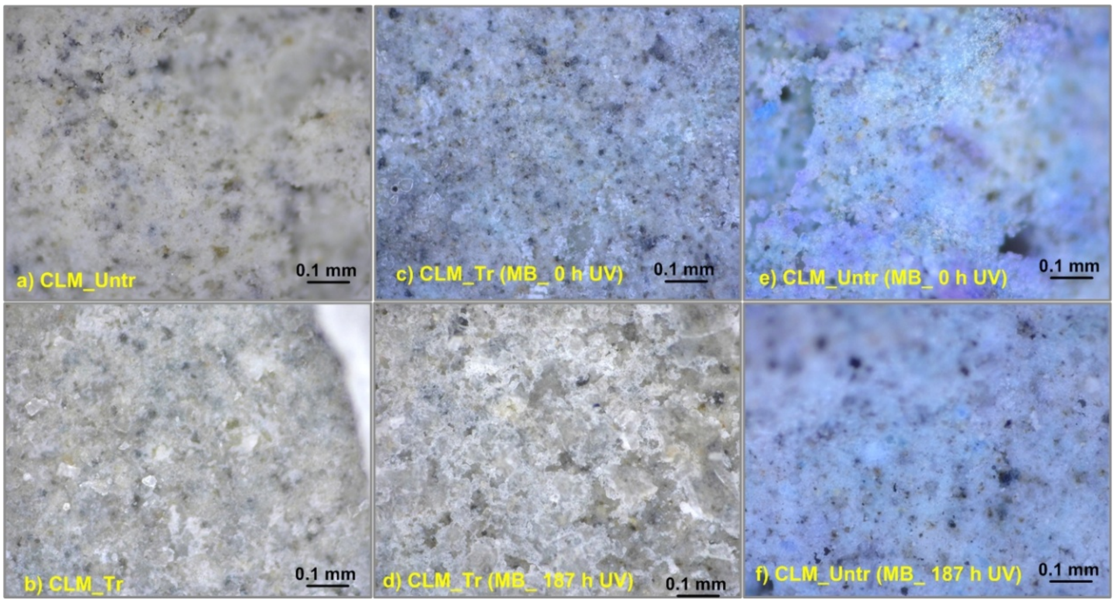

Figure S6. Optical Microscopy images of CLM substrate: untreated (a), treated with TCO&SP&T (b); treated and stained: before (c) and after (d) UV irradiation; untreated and stained: before (e) and after (f) UV irradiation.

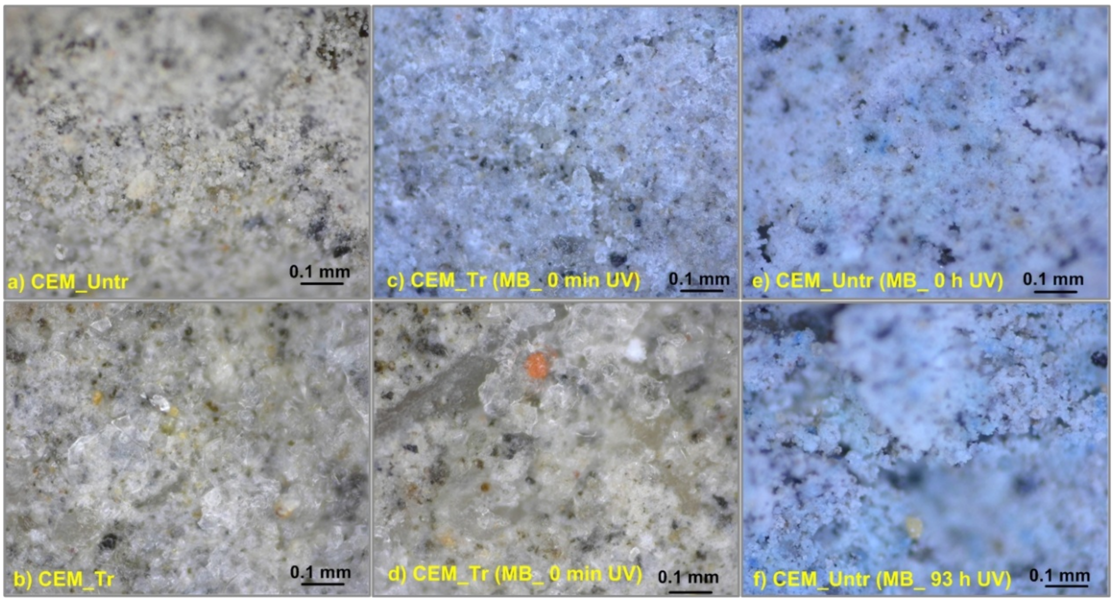

Figure S7. Optical Microscopy images of CEM substrate: untreated (a), treated with TCO&SP&T (b); treated and stained: before (c) and after (d) UV irradiation; untreated and stained: before (e) and after (f) UV irradiation.

S3. SEM Images of Untreated and Treated Substrates

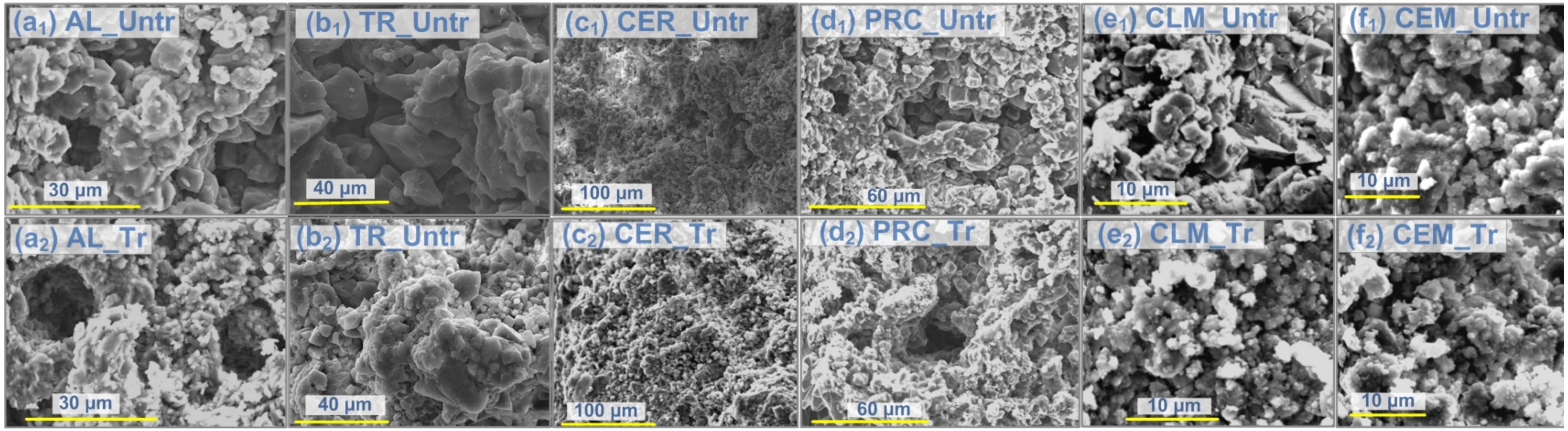

Figure S8. SEM images of untreated (a1, b1, c1, d1, e1, f1) and treated with TCO&SP&T (a2, b2, c2, d2, e2, f2) for AL, TR, CER, PRC, CLM, CEM substrates, respective
